# Optimizing Occlusal Splint Therapy: ElMohandes Protocol for Minimizing Musculoskeletal Alterations With Reduced Wear Time

**DOI:** 10.7759/cureus.88370

**Published:** 2025-07-20

**Authors:** Wael A El-Mohandes, Mahmoud S. Elariby, Ahmed Bahaa

**Affiliations:** 1 Oral and Maxillofacial Surgery, Faculty of Dental Medicine, Al-Azhar University, Cairo, EGY; 2 Oral and Maxillofacial Surgery, Benghazi University, Benghazi, LBY; 3 Implant, Oral and Maxillofacial Surgery, Royal College of Surgeons of Edinburgh, Edinburgh, GBR

**Keywords:** educational intervention, occlusal splint, physiotherapy, tens, tmds, tmj

## Abstract

Aim: The aim of this study is to evaluate the clinical efficacy and safety of the ElMohandes protocol, a combined regimen of intermittent occlusal splint therapy, musculoskeletal physiotherapy, and structured patient education, in reducing pain, enhancing jaw function, and minimizing occlusal side effects in adults with myogenic temporomandibular disorders (TMD) or internal derangement.

Methods: Forty-two consecutive patients (age 18-55 years) diagnosed with myogenous TMD or temporomandibular joint (TMJ) internal derangement according to diagnostic criteria for temporomandibular disorders (DC/TMD) were enrolled in this single-arm case series. Exclusion criteria included prior TMJ surgery, condylar fracture, systemic disease, and recent minimally invasive TMJ interventions. Each participant received a maxillary stabilization splint to be worn 10-12 hours/day over six months, with gradual “weaning in” and “weaning out” phases. Adjunctive therapy comprised thrice‑weekly sessions of transcutaneous electrical nerve stimulation (20-30 min) and ultrasound (8 MHz, 0.73 W/cm²) for four weeks, delivered by an expert physical therapist. A multimodal educational program on TMD etiology, self-management, and stress reduction was provided through face-to-face and digital materials. Primary outcomes, i.e., pain intensity (visual analog scale), maximal interincisal opening (MIO), muscle pain/tenderness scores, and patient satisfaction, were assessed at baseline, one week, and one, three, and six months. Statistical analyses included repeated‑measures ANOVA, Friedman tests, and chi-square tests (α=0.05).

Results: All 42 patients completed the protocol with no dropouts. Mean VAS pain scores declined from 7.57 ± 0.50 at baseline to 3.38 ± 0.49 at six months (p<0.001), and mean MIO increased from 31.87 ± 1.34 mm to 35.61 ± 1.70 mm (p<0.001). Significant muscle pain and tenderness improvements were observed at all post-baseline timepoints (p<0.0001). At six months, 76.2% of patients reported “highly satisfied” functional recovery, 19.0% reported “quiescent acceptance,” and 4.8% remained “unsatisfied” (p<0.0001). No clinically relevant occlusal changes or adverse events were detected.

Conclusion: The ElMohandes protocol yielded rapid, substantial reductions in pain and functional gains while preserving occlusal stability and achieving high patient satisfaction. Its integration of part-time splint use, physiotherapeutic modalities, and patient education appears to optimize conservative TMD management. Randomized controlled trials with objective compliance monitoring and extended follow-up are warranted to confirm these findings and establish long-term benefits.

## Introduction

Temporomandibular disorders (TMD) are multifactorial conditions of the temporomandibular joint (TMJ) and masticatory muscles that cause pain, limited jaw motion, and tenderness, substantially impairing quality of life [1]. Understanding these complex etiologies is essential to designing effective, targeted therapies. Given the multifactorial origins of TMD, conservative, low-risk interventions are often first‑line for TMD, including physical therapy (e.g., jaw exercises), behavioral modification, and adjunctive pharmacotherapy (analgesics, muscle relaxants), which is widely adopted for its safety and patient acceptability [2]. Among these, occlusal splint therapy is prevalent.

Occlusal splint therapy is another widely used non-invasive treatment for TMD. These devices, known as bite splints or night guards, are designed to reduce muscle hyperactivity and protect the TMJ from excessive forces. Studies from a systematic review have demonstrated that occlusal splints can alleviate pain and improve jaw function [3]. The selection of splint type and the duration of use are crucial factors in achieving optimal therapeutic outcomes [3]. Customization and patient compliance play significant roles in the success of this treatment modality [3]. However, the ideal daily wear time remains debated.

The duration of occlusal splint use varies among patients, depending on the severity of symptoms and individual response to treatment. Recommended wear times range from 8-12 hours nightly, with some protocols extending to daytime use for patients with prominent daytime symptoms [4-6]. Daytime use can help manage acute pain episodes and prevent further damage to the TMJ. The combined use of the splint during both day and night may enhance the overall effectiveness of the treatment. Regular follow-up with a healthcare provider is essential to monitor progress and make necessary adjustments to the treatment plan [7].

Despite extensive evidence showing that occlusal splint therapy effectively relieves TMD symptoms, certain types of splints, especially those used full-time or without proper occlusal design, have been linked to musculoskeletal and occlusal side effects, such as mandibular displacement and unstable occlusion. These adverse effects are often reported with poorly adjusted or non-anatomical appliances and are less familiar with carefully fabricated designs like the Michigan splint. This distinction highlights the importance of splint type, duration of use, and proper adjustment in minimizing iatrogenic effects and optimizing therapeutic outcomes. The existing literature primarily emphasizes the benefits of prolonged splint wear, typically 8-12 hours nightly or continuously, while providing limited guidance on strategies to prevent these adverse outcomes [3,4,8-10].

To mitigate these potential side effects, our study introduces the ElMohandes protocol, adjusting the number of hours of splint use to intermittent 10-12 hours to prevent musculoskeletal changes that could arise from 24-hour daily use in TMD patients. This strategy, combined with targeted physiotherapy and comprehensive patient education, aims to preserve therapeutic benefits while reducing potential complications.

## Materials and methods

To test this protocol, we conducted a prospective case series study involving 42 consecutive patients with TMD recruited from the Outpatient Clinic of Oral and Maxillofacial Surgery, Faculty of Dental Medicine, Al-Azhar University, Cairo, Egypt, and Sayed Galal University Hospital. Eligibility criteria are shown in Table 1. The study was approved by the Research Ethics Committee of the Faculty of Dental Medicine, Al-Azhar University, with approval code 1128/79. All the eligible patients signed an informed consent form and received a detailed explanation of the treatment protocol. The study started from October 2024 and ended in April 2025.

**Table 1:** Eligibility criteria. TMJ: temporomandibular joint, TMD: temporomandibular disorder.

Inclusion criteria	Exclusion criteria
Patients aged 18 to 55 years.	Prior surgical operations related to TMJ.
Diagnosed with myogenous TMDs or TMJ internal derangement according to the Diagnostic Criteria for Temporomandibular Disorders [11]	History of fractured condyle.
	Previous minimally invasive treatments for TMJ.
	Immunocompromised status.
	Hematological disorders.
	Pregnancy

Upon enrollment, participants provided personal, medical, and dental histories. Their personal information was recorded, and medical histories were reviewed to exclude systemic diseases. A comprehensive dental evaluation, including clinical and radiographic, was conducted to rule out any odontogenic pain, missing molars, and occlusal abnormalities like bruxism and deep bite. The preoperative assessment included a comprehensive clinical examination based on the temporomandibular disorders (DC/TMD). Key components of this evaluation involved a detailed questionnaire capturing personal data, medical history, previous treatments, and the chief complaint. Pain levels were measured using a visual analogue scale (VAS), with patients rating their pain from 0 (no pain) to 10 (unbearable pain). Furthermore, maximum interincisal opening (MIO) was recorded with a digital caliper.

**Figure 1:**
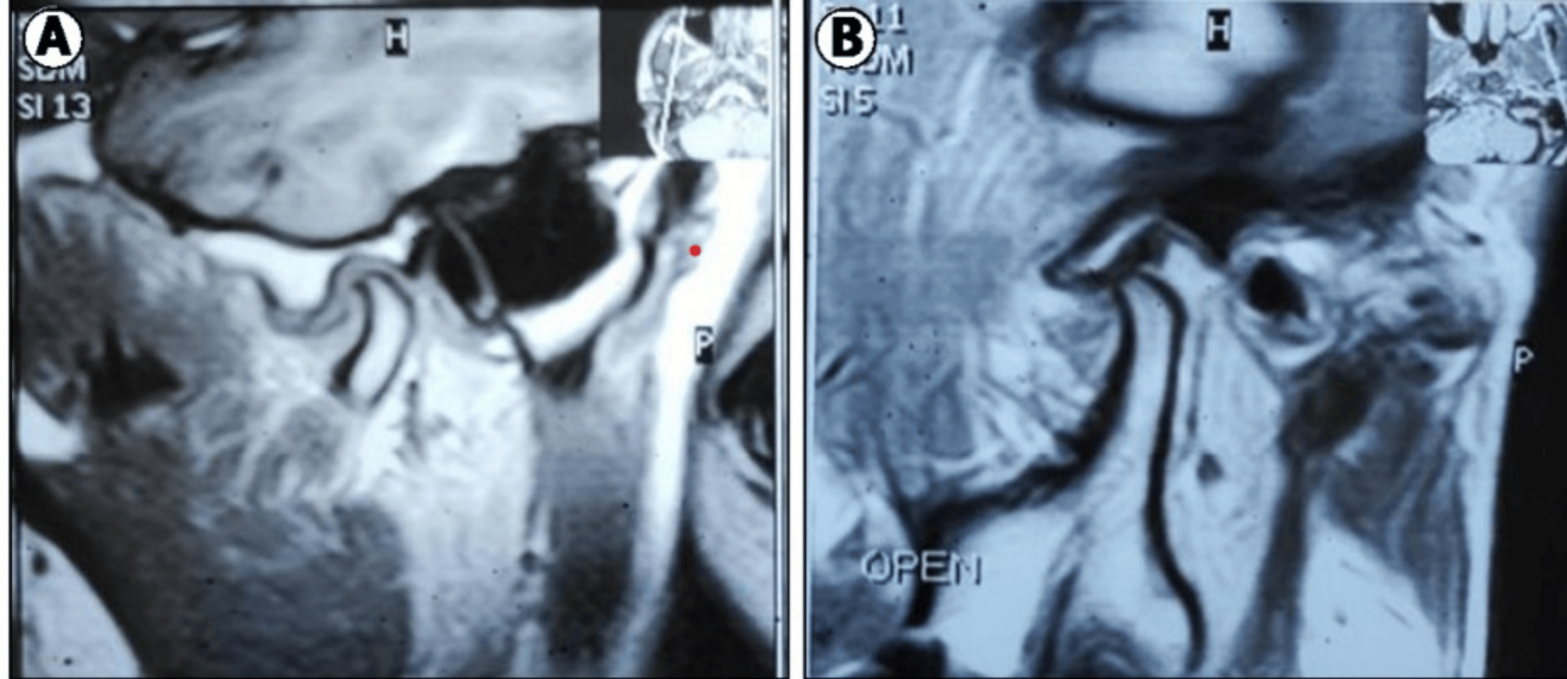
Preoperative MRI images. A: Closed mouth; B: open mouth

Additional physical examinations focused on joint noises, muscle pain (specifically in the masseter and temporalis muscles), and external joint tenderness. The TMJ was evaluated bilaterally using a structured palpation technique to identify tenderness and structural anomalies. Finally, radiographic evaluation via magnetic resonance imaging (MRI) was conducted for all patients to detect abnormalities in disc form and position in various mouth positions, ensuring a thorough preoperative assessment to inform treatment planning (Figure 1).

ElMohandes protocol

**Figure 2:**
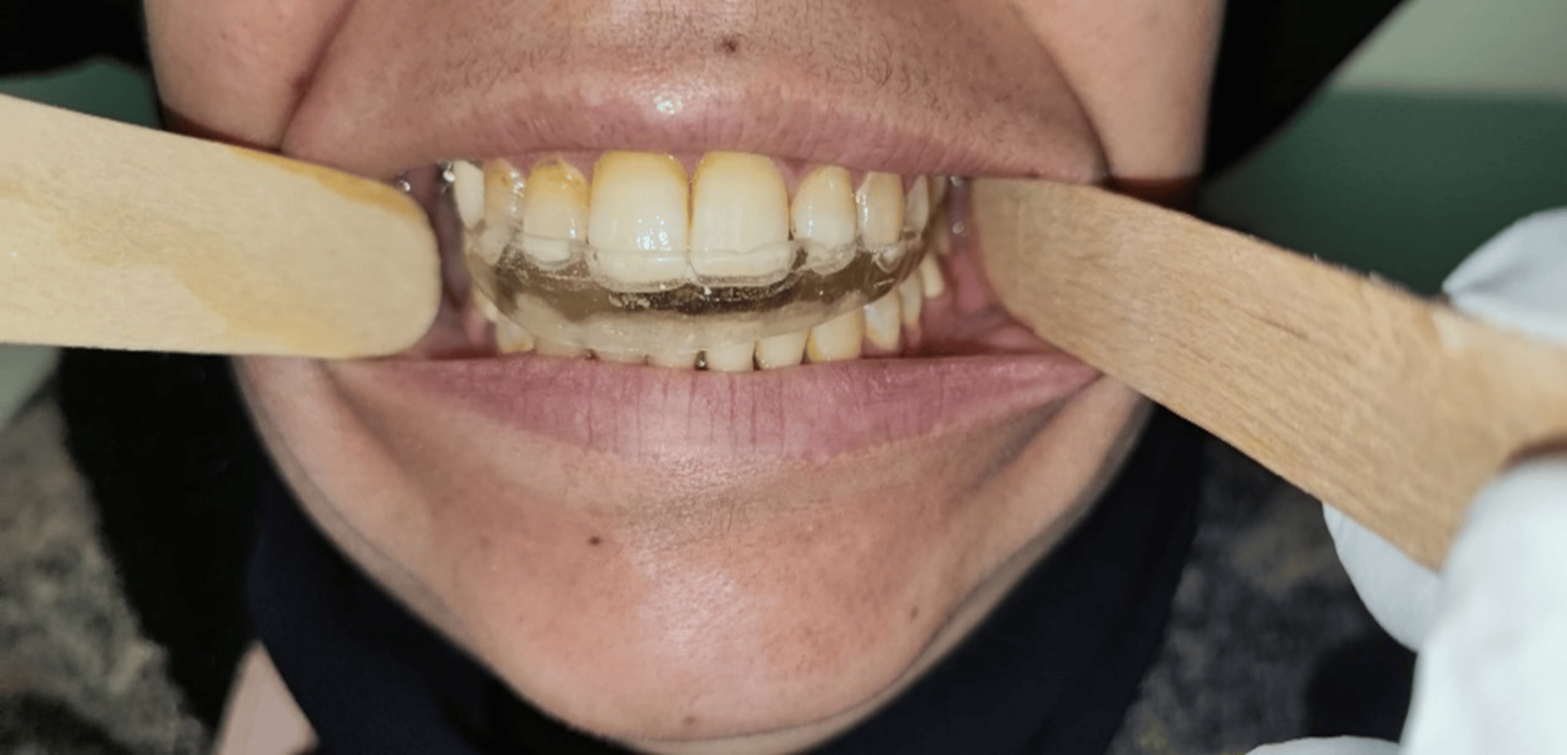
Occlusal splint adapted to the maxillary teeth and supported by an Adams clasp.

**Figure 3:**
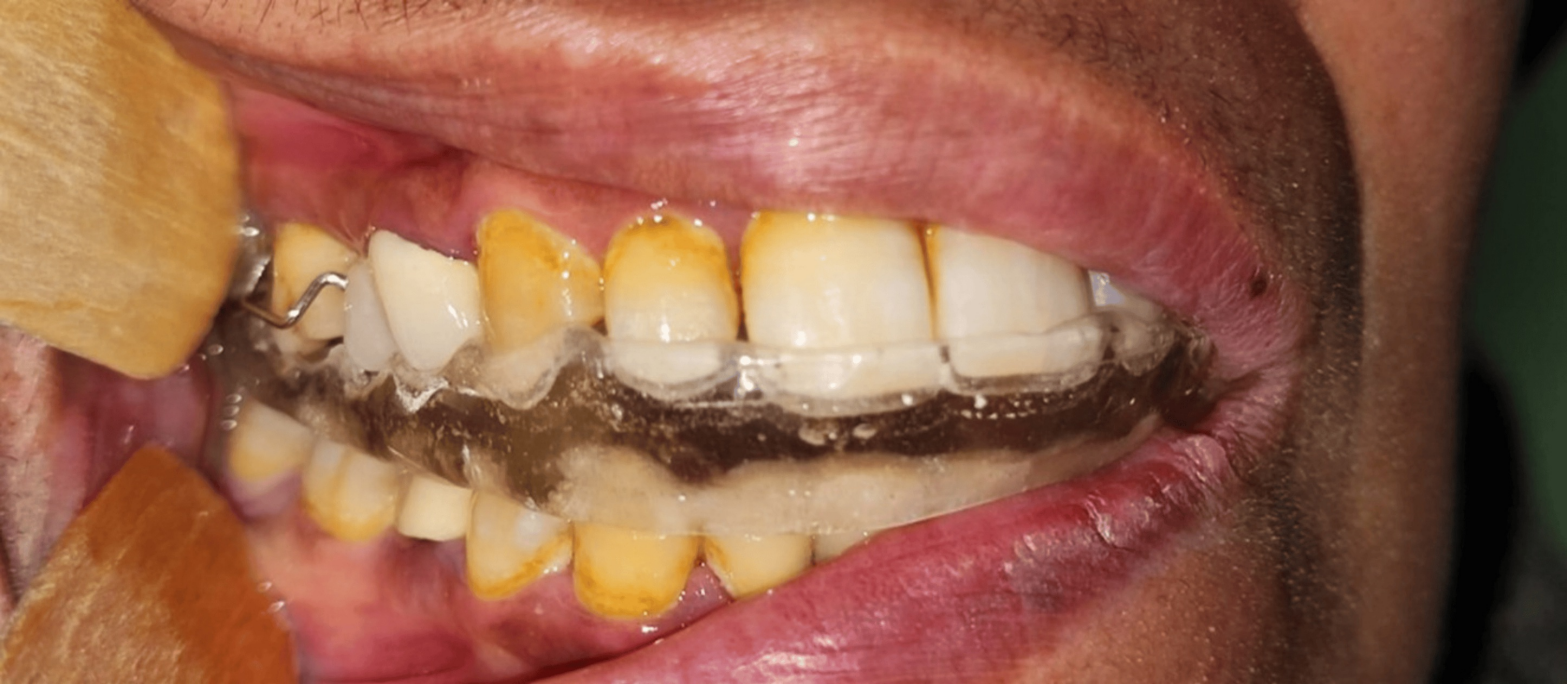
Right lateral view with an occlusal splint adapted to the maxillary teeth.

**Figure 4:**
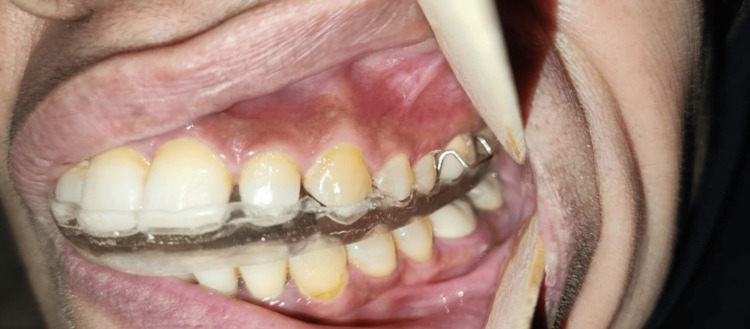
Left lateral view with an occlusal splint adapted to the maxillary teeth.

All participants received a maxillary splint. The maxillary splint used in this study was the stabilization/Michigan splint, consisting of a rigid splint constructed for the maxillary arch, including all maxillary teeth, with a flat occlusal plane. A researcher took accurate alginate impressions of both arches and obtained an interocclusal wax wafer registration for all participants. After one week, participants received the occlusal splint, which was appropriately adjusted in centric occlusion, made with methyl methacrylate acrylic, and had a maximum occlusal thickness of 3 mm. The ElMohandes protocol of splint therapy consisted of daily use of the splint for 10-12 hours over six months (with weaning in and weaning out) along with specific educational instructions and musculoskeletal physiotherapy (Figures 2-4).

During the study, no participants received any other form of treatment, including drugs or occlusal adjustments, except a muscle relaxant for the first week. The researchers conducted the occlusal splint and educational intervention, whereas an orthopedic manipulative physical therapist, an expert in this field, performed musculoskeletal physiotherapy treatment.

The musculoskeletal physiotherapy treatment included a combination of ultrasound application and transcutaneous electrical nerve stimulation (TENS), which was provided three times a week for four weeks. TENS therapy delivers low-voltage, pulsed electrical currents (low frequency current < 10Hz, 40 to 75 microseconds pulse) to alleviate pain and relax muscles associated with the TMJ and masticatory muscles for 20-30 minutes per session [12]. Therapeutic ultrasound was applied using a SIEMENS ACUSON diagnostic unit with an 8 MHz probe at an intensity of 0.73 ± 0.084 W/cm², targeting the masseter muscle and mandibular ramus [13].

The educational materials provided a thorough overview of TMDs, addressing their causes, such as muscle tension, joint dysfunction, stress, and parafunctional habits. They also outlined symptoms such as jaw pain, clicking, and limited movement. The educational program focused on self-management techniques through lifestyle changes, pain relief strategies, and correct posture. The program utilized a multimodal approach, including face-to-face interactions, printed resources, and digital media, to cater to various learning styles and reinforce essential concepts. Initial sessions focused on diagnosis and treatment options, including occlusal splints, ultrasound parameters, and TENS, lasting 30 to 60 minutes. Follow-up sessions were shorter (15-30 minutes) over 2 to 6 weeks, with weekly educational reinforcement in early treatment, transitioning to biweekly or monthly as patients gained self-efficacy.

Outcomes

The primary outcomes assessed were postoperative pain, MIO, muscle pain, tenderness, and patient acceptance of the treatment. Postoperative pain, MIO, muscle pain, and tenderness were assessed at baseline, after one week, and after one, three, and six months. Postoperative pain was evaluated using a 0-10 cm VAS. MIO was measured using a digital caliper. Muscle pain and tenderness were assessed using a categorical scale of 0 to 4 [14,15]. The categories for muscle pain included none, spontaneous, mild, moderate, and severe, while the categories for muscle tenderness comprised none, mild, moderate, severe, and withdrawal to touch. Patient acceptance was evaluated through a questionnaire completed by the patient and recorded by the examiner after six months. This questionnaire described how patients were able to return to their everyday lives on a three-category scale: Highly satisfied (symptoms of TMDs disappeared) patients returned to normal function and lifestyle; Quiescent acceptance (symptoms markedly improved but did not disappear) patients experienced improved function and lifestyle but did not return to normal; and Unsatisfied (symptoms still exist or exaggerated) patients could not return to normal function or lifestyle [16].

Statistical methods

The significance level was set at 5%. The data was analyzed using IBM SPSS Statistics for Windows, Version 22 (Released 2013; IBM Corp., Armonk, New York, United States). Continuous data was summarized into mean and standard deviation, while categorical data was summarized into frequency and proportions. Data were checked for normality using the Shapiro-Wilk test. 95% confidence intervals were calculated. One-way repeated-measure ANOVA was used to compare mean values among different time points for parametric data. In case of a significant ANOVA, multiple paired t-tests with Bonferroni multiple testing correction were conducted to identify significant pairwise comparisons. Friedman test, followed by the Wilcoxon test and Holm correction, was used to compare mean values for different time points in the case of non-parametric data and ordinal categorical data with more than three levels. The chi-square goodness of fit test was used to compare proportions for categorical ordinal data with three levels.

## Results

All 42 patients completed the six-month follow-up with no dropouts. All the data showed a non-normal distribution, as evident by a statistically significant Shapiro-Wilk test (p < 0.05) except for the normally distributed MIO data (p = 0.657).

Table 2 shows results for the postoperative pain intensity and MIO values at different follow-up periods. Both the postoperative pain intensity and MIO values showed statistically significant reduction in pain and MIO through time. The post hoc tests for pairwise comparisons (Wilcoxon with Holm correction and multiple paired t-tests with Bonferroni correction) indicated statistically significant differences between different pairwise timepoint comparisons in each outcome (p < 0.05), except for the one week and one month comparison in the pain scores (p = 0.054) and the baseline and one month comparison in the MIO values (p = 0.307).

**Table 2:** Results for postoperative pain intensity and MIO values during different follow-up periods. MIO: maximal interincisal opening, SD: standard deviation, *: statistically significant difference. $: p-value acquired from the Friedman test, &: p-value acquired from the repeated measure ANOVA test. The total number of patients at each follow-up time was 42.

Variable	Postoperative pain intensity		MIO values (cm)	
	Mean	SD	Mean	SD
Baseline	7.57	0.50	31.87	1.34
One week	6.19	0.63	30.34	1.81
One month	5.86	0.75	32.14	1.44
Three months	5.40	0.50	33.76	1.52
Six months	3.38	0.49	35.61	1.70
p-value	< 0.001*^$^		< 0.001*^&^	

**Figure 5:**
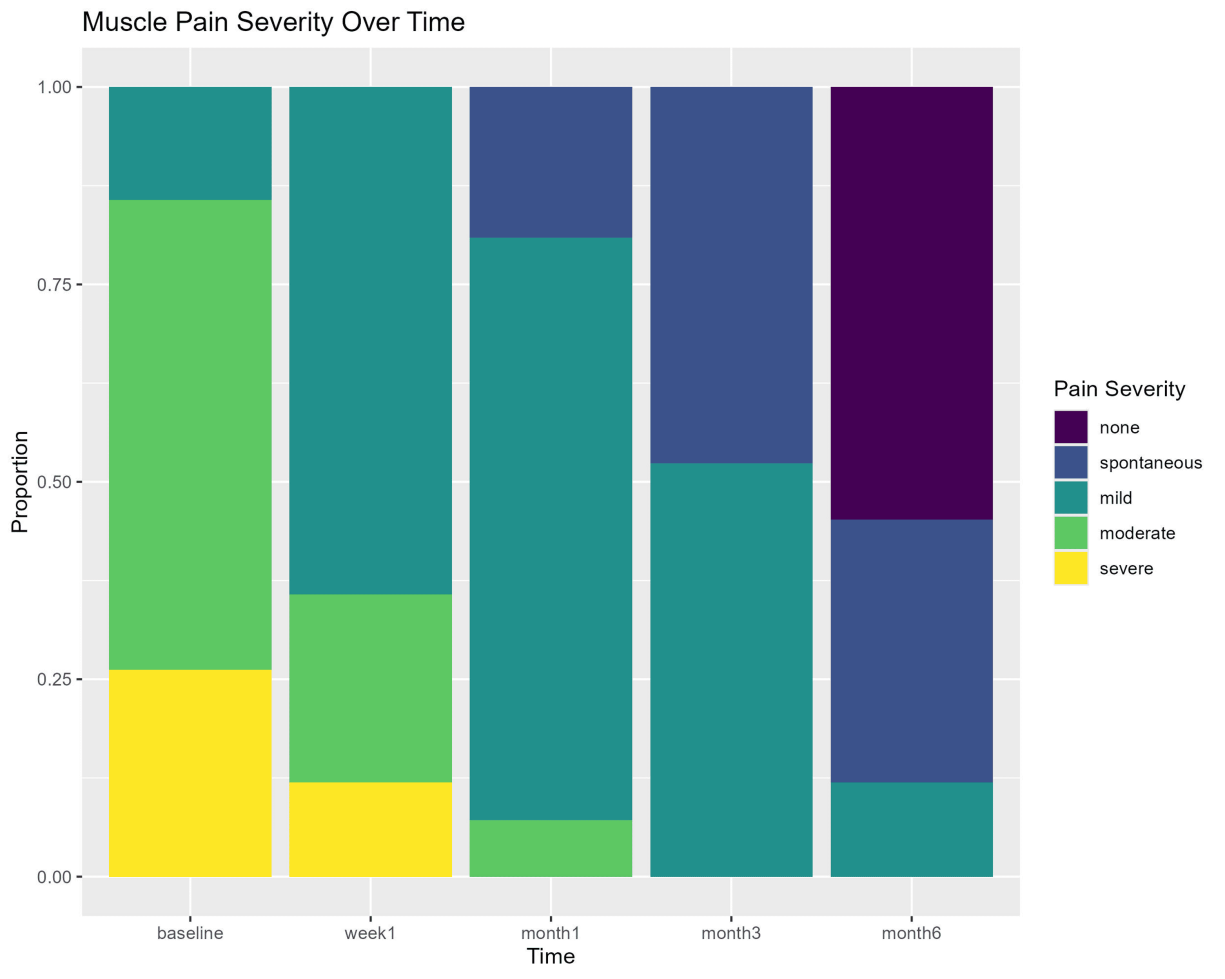
Muscle pain severity over time stacked bar chart.

**Figure 6:**
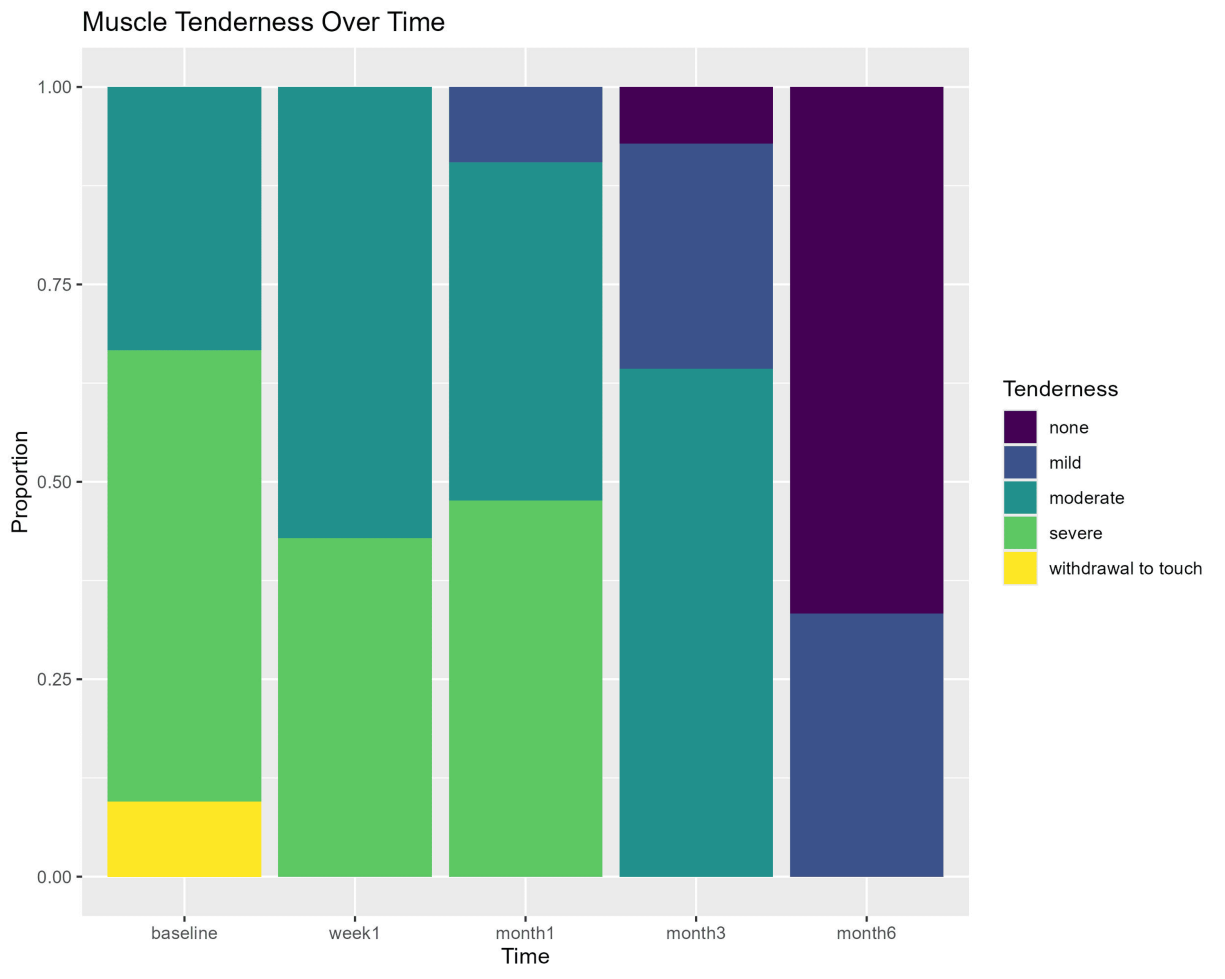
Muscle tenderness over time stacked bar chart.

**Figure 7:**
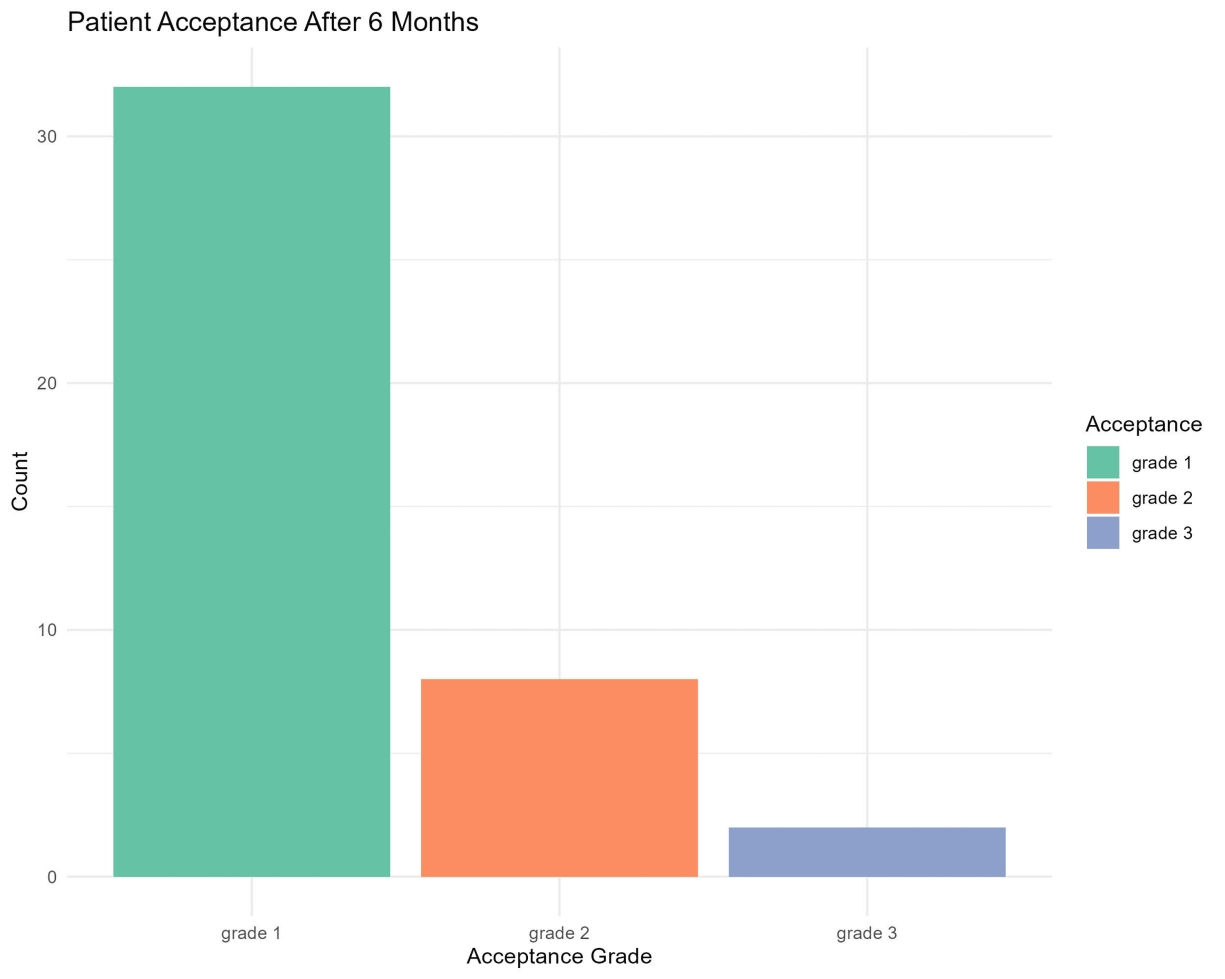
Bar chart showing patient acceptance for the treatment after six months.

Tables 3-5 show the percentage of muscle pain severity, tenderness, and patient acceptance to treatment for all time points assessed. Muscle pain severity and tenderness showed statistically significant improvement over time (p < 0.00001). Wilcoxon test pairwise comparisons (with the Holm correction) showed a statistically significant difference in all pairwise comparisons (p < 0.0004) except for the one-week and one-month comparison in the muscle tenderness outcome (p = 0.484). Figures 5-7 show a graphic presentation of the proportions of muscle pain severity, muscle tenderness, and patient acceptance of treatment. The graphs show improvement of the severity of muscle pain and tenderness through time, with 54.8% and 66.7% showing no pain and tenderness after six months. Patient acceptance of treatment after six months showed statistically significant differences using the chi-square test (p < 0.0001), with 76% of the patients being highly satisfied.

**Table 3:** Muscle pain severity scores and percentages at different timepoints. * Statistically significant differences were obtained from Friedman's test. The total number of patients at each follow-up time was 42.

Timepoint	None	Spontaneous	Mild	Moderate	Severe	p-value
Baseline	0	0	6 (14.3%)	25 (59.5%)	11 (26.2)	< 0.00001*
One week	0	0	27 (64.3%)	10 (23.8%)	5 (11.9%)	
One month	0	8 (19%)	31 (73.8%)	3 (7.1%)	0	
Three months	0	20 (47.6%)	22 (52.4%)	0	0	
Six months	23 (54.8%)	14 (33.3%)	5 (11.9%)	0	0	

**Table 4:** Muscle tenderness scores and percentages at different timepoints. * Statistically significant differences were obtained from Friedman's test. The total number of patients at each follow-up time was 42.

Timepoint	None	Mild	Moderate	Severe	Withdrawal to touch	p-value
Baseline	0	0	14 (33.3%)	24 (57.1%)	4 (9.5%)	< 0.00001*
1 week	0	0	24 (57.1%)	18 (42.9%)	0	
1 month	0	4 (9.5%)	18 (42.9%)	20 (47.6%)	0	
3 months	3 (7.1%)	12 (28.6%)	27 (64.3%)	0	0	
6 months	28 (66.7%)	14 (33.3%)	0	0	0	

**Table 5:** Results for patient acceptance for treatment after six months. * Statistically significant differences were obtained from the chi-square goodness of fit test. The total number of patients was 42.

Timepoint	Grade 1	Grade 2	Grade 3	p-value
6 months	32 (76.2%)	8 (19%)	2 (4.8%)	< 0.00001*

## Discussion

Diagnosis and treatment of TMJ remain a challenge, and there is still no consensus on many aspects. The most widely accepted and standardized tool for the assessment and classification of TMD is DC/TMD, which contains a structural Axis-I with a prescribed physical examination protocol to make a specific physical diagnosis of TMD concerning the joints and muscles and a biopsychosocial component. Axis II includes several tools to assess the patient's psychological state [11]. Various conservative treatment options have shown that using occlusal splints reduces pain intensity and has a positive effect. Occlusal splint therapy has emerged as a cornerstone in the management of TMDs and bruxism, with extensive literature attesting to its efficacy in symptom alleviation and musculoskeletal stabilization [3].

In this single-arm case series involving 42 patients with myogenous TMD or internal derangement, the ElMohandes protocol, which includes 10 to 12 hours per day of intermittent stabilization splint wear, thrice-weekly TENS and ultrasound therapy, and structured patient education, yielded significant clinical improvements. After six months, mean pain scores (VAS) decreased by 55.35% (mean difference -4.19, 95% CI -3.95 to -4.42), and MIO increased by 3.7 mm (mean difference 3.74, 95% CI 4.31 to 3.17). Additionally, 76.2% of participants reported being "highly satisfied" with their functional recovery, and no significant occlusal changes or adverse events were noted. These results are favorable compared to conventional splint protocols, indicating that the combined approach provides effective symptomatic relief and functional enhancements while maintaining occlusal stability [3-7].

Occlusal splint therapy serves as a fundamental approach in conservative management for TMD. Typically, wearing a splint full-time or nightly leads to a 50-65% reduction in pain over 8-12 weeks [3]. However, prolonged use can result in undesired musculoskeletal changes, including mandibular displacement, and alterations in occlusion, such as changes in occlusal contacts and vertical dimension [4,6,7]. Part-time or intermittent wear (at least eight hours per night) has been explored to address these concerns. Luther et al. (2010) found that wearing a splint for eight hours each night provided similar pain relief while decreasing the chance of occlusal shifts compared to full-time use [17]. Other studies by Tăut et al. (2024) and Amuthan & Gayathri (2023) indicated that intermittent splint use combined with manual therapy resulted in significant condylar remodeling and reduced oxidative stress, respectively [18,19].

Our findings build on previous observations by integrating electrical stimulation and ultrasound therapies. Meta-analyses suggest that combining splint therapy with physiotherapeutic interventions can enhance pain relief by an additional 20-30% and accelerate functional recovery [20]. In our study, incorporating TENS and ultrasound led to 64% of patients reporting only mild pain within one week, significantly faster than splint-only studies, which typically report substantial relief after 2 to 4 weeks [21,22].

Occlusal splints are thought to enhance the redistribution of occlusal forces, lessen parafunctional muscle hyperactivity, and stabilize the TMJ by modifying neuromuscular patterns and occlusal contacts [7,16,23]. The ElMohandes protocol splint, crafted from hard acrylic and adjusted for uniform contacts, optimizes joint loading and diminishes masticatory muscle hyperactivity. TENS induces analgesia by activating enkephalinergic and β‐endorphin pathways, which elevate pressure‐pain thresholds and reduce electromyographic activity in the masseter and temporalis muscles [24]. Therapeutic ultrasound promotes tissue heating, increases local blood flow, and facilitates myofascial release, resulting in better joint mobility without significant differences between continuous and pulsed modes [22]. The synergistic application of these modalities is likely the reason for the accelerated symptom resolution observed.

The ElMohandes protocol’s intermittent splint usage helps maintain occlusal stability, as suggested by Manfredini et al., who advise against full-time use to prevent potential occlusal side effects [25]. Implementing adjunctive therapies three times a week not only accelerates pain relief but may also decrease dependence on pharmacologic analgesics, thereby reducing the risk of medication-related adverse events. Additionally, the structured education component, featuring stress management and self-care techniques, seems to enhance patient adherence and self-efficacy, resulting in a notably low relapse rate of 19% at six months. This figure is significantly better than the 30-40% recurrence rates often seen with unsupervised splint use [26,27].

While the outcomes are encouraging, several limitations affect the broader applicability of our findings. First, the study's single-arm case series design lacks a randomized control group, preventing the establishment of definitive causal relationships between the intervention and outcomes. Additionally, the follow-up lasted only six months, providing insights into short- to mid-term efficacy but not addressing long-term relapse rates or occlusal stability beyond one year. Moreover, the study focused solely on myogenous and internal derangement cases according to DC/TMD criteria, potentially overlooking how arthrogenous or mixed-type TMD presentations might respond to the treatment. Finally, compliance was measured through self-reported data on wear time and home exercises, which may lead to an overestimation of actual adherence due to the lack of objective monitoring [28]. These limitations underscore the need for controlled trials to validate the intervention's efficacy and safety in various contexts.

We propose priority research areas to enhance the understanding and treatment of TMD. Key recommendations include conducting randomized controlled trials to compare intermittent and full-time splint wear, with and without physiotherapy, and using objective monitoring tools like sensor-equipped splints or mobile apps to track compliance. Long-term follow-ups at 12, 18, and 24 months are vital to assess stability, relapse rates, and late-onset changes. We also support TMD subtype stratification to evaluate treatment applicability across different diagnoses. Cost-effectiveness analyses will provide insights into resource use and improve clinical guidelines. Finally, investigating new therapies such as low-level laser therapy and cognitive-behavioral interventions could enhance patient-reported outcomes. These efforts aim to optimize treatment plans and personalize care for TMD patients.

## Conclusions

In conclusion, the investigations into TENS and ultrasound therapies reveal their significant role in managing TMD, providing substantial analgesic and functional improvements with minimal side effects. Similarly, ElMohandes’s innovative protocol for occlusal splint therapy marks a substantial advancement by enhancing treatment effectiveness and reducing negative outcomes. To fully establish the clinical relevance of these approaches, future studies should focus on conducting comprehensive randomized controlled trials that involve diverse patient populations, extended follow-up periods, and rigorous compliance monitoring. These strategies will affirm the benefits of combining these therapies and protocols in enhancing patient care and outcomes.

## Appendices

Questionnaire

https://docs.google.com/document/d/1BKlHc36tI5KqW2km7wtwJ6mq52ECTruC/edit?usp=sharing&ouid=111253333842660597349&rtpof=true&sd=true
